# The Association between Serum Total Bile Acid Level and Long-Term Prognosis in Patients with Coronary Chronic Total Occlusion Undergoing Percutaneous Coronary Intervention

**DOI:** 10.1155/2022/1434111

**Published:** 2022-06-23

**Authors:** Xinchun Zeng, Zhijie Jian, Shanshan Li, Yu Xu, Bolin Li, Ning Ding, Yiqiong Zhang, Hui Zhang, Yue Wu, Jian Yang, Zuyi Yuan, Lele Cheng

**Affiliations:** ^1^Department of Cardiovascular Medicine, The First Affiliated Hospital, Xi'an Jiaotong University, Xi'an, Shaanxi, China; ^2^Department of Medical Imaging, The First Affiliated Hospital, Xi'an Jiaotong University, Xi'an, Shaanxi, China; ^3^Key Laboratory of Molecular Cardiology, Shaanxi Province, Xi'an, Shaanxi, China; ^4^Key Laboratory of Environment and Genes Related to Diseases, Ministry of Education, Xi'an, Shaanxi, China

## Abstract

**Background and Aims:**

Bile acids, the key products for elimination of cholesterol, play an important role in coronary artery disease (CAD). However, few studies focused on the roles of more accessible serum total bile acids (TBA) in the prediction of adverse cardiovascular events for coronary chronic artery occlusion (CTO). The aim of this study was to explore the potential relationship between serum TBA and long-term prognosis in patients with CTO undergoing percutaneous coronary intervention (PCI).

**Methods:**

Baseline TBA was determined in 613 patients with CTO after PCI in the present study. All patients were divided into 3 groups according to the median (3.5 *μ*mol/l) and the normal upper limit of the TBA (10 *μ*mol/l). The primary endpoint was all-cause mortality, and the secondary endpoint was major adverse cardiovascular events (MACE).

**Results:**

Average age in this study was 65.44 ± 9.94 years old. The median of TBA was 3.5 (2.1-6.1) *μ*mol/l. Over a median follow-up of 33.5 months, compared to those with below 3.5 *μ*mol/l TBA, 3.5 ~ 10 *μ*mol/l TBA was associated with significantly reduced risk for the MACE (hazard ratio (HR): 0.59, 95% confidence interval (CI): 0.40 to 0.88; *p* = 0.009) even after adjustment for baseline variables. However, TBA did not predict all-cause mortality and cardiovascular death. Spline analyses showed an L-shaped relationship of the serum TBA with the incidence of MACE.

**Conclusions:**

Moderate fasting serum TBA level has a predictive value for MACE even after adjusting for lifestyle and clinical risk factors in CTO patients undergoing PCI.

## 1. Introduction

Bile acids (BAs) are amphipathic end products of cholesterol metabolism, which function both as detergents to facilitate the digestion and absorption of dietary lipids, and as hormones with systemic endocrine functions [1]. Formed in the liver and stored in the gall bladder, BAs are absorbed actively from the small intestine, with each molecule undergoing multiple enterohepatic circulations before being excreted. The primary BAs are metabolized by the gut microbiota to generate the secondary BAs [2]. In addition, BAs are conjugated to glycine or taurine to form conjugated BAs in liver for biliary secretion [3]. The synthesis of BAs is an important pathway for elimination of cholesterol from body. Studies suggested that BA sequestrants are one of the strategies for regulating blood cholesterol levels and atherosclerotic plaque formation [4]. Hence, it is of great clinical importance to explore the association between serum total BA level and coronary artery disease (CAD).

Several studies have demonstrated the intrinsic links between myocardial infarction and BAs through cholesterol metabolism [5]. And our previous cross-sectional study found that fasting serum total bile acid (TBA) level is highly associated with the presence and severity of CAD in patients undergoing coronary angiography for suspected CAD [6]. However, the relationship between TBA and long-term adverse cardiovascular events in patients with coronary chronic artery occlusion (CTO) undergoing percutaneous coronary intervention (PCI) remains largely unclear. CTO lesions are commonly encountered during coronary angiography, with the higher risk for adverse cardiovascular outcomes and lower success rate of PCI than non-CTO lesions in patients with CAD [7, 8]. Thus, the present study was performed to determine and investigate whether the TBA has a predictive value for long-term adverse cardiovascular events in patients with CTO after PCI.

## 2. Methods

### 2.1. Patient Characteristics

**Figure 1:**
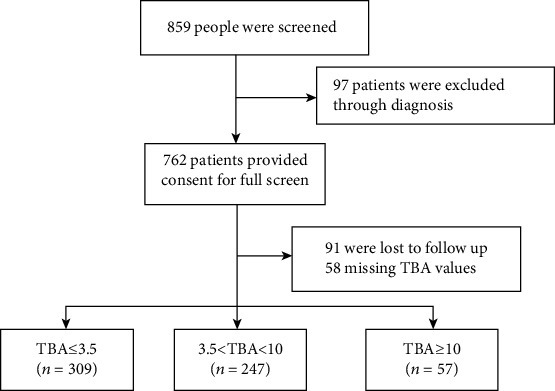
Study flow for the present analysis. A total of 859 patients were screened initially, and 762 patients were performed CTO-PCI. We excluded 91 patients who were lost in follow-up, 58 patients whose TBA values were missed, and finally 613 patients were included in this study.

We reviewed the files of CTO patents in the First Affiliated Hospital of Xi'an Jiaotong University from June 2013 to October 2017. A total of 859 patients were screened initially, and 762 patients were performed CTO-PCI. We excluded 91 patients who lost follow-up, 58 patients whose TBA values were missed, and finally 613 patients were included in this study (Figure 1). The inclusion criteria and exclusion criteria were as previously described [9]. The detailed demographic, clinical, drug, and hematologic data were obtained from the medical records. In addition, we collected 7829 patients who had the values of TBA from the Biobank in the First Affiliated Hospital of Xi'an Jiaotong University to study the association between TBA and myocardial infarction and cerebral infarction. The patients were inpatients or outpatients of Neurology or Cardiovascular Medicine Department in this cross-sectional study. Our study was conducted according to the Declaration of Helsinki and was approved by the Ethics Committee of the First Affiliated Hospital of Xi'an Jiaotong University. Written informed consent was obtained from all study participants.

### 2.2. Biochemical Measurements

Fasting peripheral blood was sampled from patients within 24 h of admission prior to stent implantation. Serum TBA was measured with an enzymatic cycling method using reagents (Pureauto S TBA, Sekisui Medical Co., Ltd.) on a Hitachi Chemistry Analyzer 7600 according to the protocols. The normal upper limit of serum TBA was 10 *μ*mol/l. Quality control procedures were conducted according to the protocols as well, and the coefficients of variation were all ≤5%. Triglycerides (TG), low-density lipoprotein cholesterol (LDL), high-sensitivity C-reactive protein (hs-CRP), creatinine, and pro-B type natriuretic peptide (pro-BNP) were assayed at the biochemistry center of our hospital by using standard techniques. Echocardiographs were performed on admission by experienced echo cardiologists, and systolic function was expressed as the left ventricular ejection fraction (LVEF).

### 2.3. Follow-Up and End Points

All patients were followed up by interview or telephone in our hospital. Clinical outcomes were classified into the primary and secondary endpoints. The primary endpoint was all-cause mortality, and the secondary endpoint was major adverse cardiovascular events (MACE), including all-cause death, nonfatal acute myocardial infarction, revascularization, and stroke.

### 2.4. Statistical Analysis

The reference value was set at a concentration of 3.5 *μ*mol/l and 10 *μ*mol/l, which was the median concentration of TBA in the whole population and the normal upper of TBA, respectively. Then, we divided all the patients into three groups according to the TBA concentrations (≤3.5 *μ*mol/l, which was used as the reference group, 3.5 ~ 10 *μ*mol/l and ≥10 *μ*mol/l). Clinical characteristics at baseline examination were compared using *χ*^2^ tests for categorical variables and analysis of variance (ANOVA) for normally distributedcontinuous variables, the Kruskal-Wallis analysis for nonnormally distributed continuous variables.Multiple comparison, and Bonferroni correction were performed on the variables that are significantly different between the three groups. Continuous variables are presented as the mean ± standard deviation if normally distributed or median (lower quartile, upper quartile) otherwise.

A Kaplan-Meier curve was then applied to compare the prognosis between the 3 subgroups based on the reference value. The prognostic value of TBA was assessed by univariate and multivariate Cox proportional hazard models. Variables with univariate significance were selected for multivariate analysis. Survival analysis were performed using R package ‘survival' (https://CRAN.R-project.org/package=survival) and ‘survminer' (https://CRAN.R-project.org/package=survminer).

Restricted cubic splines were applied using the R package ‘rms' (https://CRAN.R-project.org/package=rms) to explore the relation of the TBA value with the HR of all-cause death, cardiovascular death, and MACE adjusted by potential confounding factors, including age, sex, smoking, hypertension, diabetes mellitus, heart rate, creatinine, revascularization, LVEF, pro-BNP, cholesterol, and triglyceride.

For the cross-sectional data, we used logistic regression analysis to study the relationship between TBA and myocardial infarction and cerebral infarction. Spearman correlation coefficients were calculated to assess the association between continuous nonnormally distributed blood lipid variables.

A *p* value < 0.05 was regarded as statistically significant for all statistical tests. All statistical analyses were performed using R version 4.0.2 software (Vienna, Austria).

## 3. Results

### 3.1. Baseline Characteristics

Baseline patient characteristics are showed in Table 1. The reference value was set at a concentration of 3.5 *μ*mol/l, which was the median concentration of TBA in the whole population. The normal upper limit of serum TBA is 10 *μ*mol/l. Therefore, all patients were segregated into three groups according to the reference value 3.5 *μ*mol/l and 10 *μ*mol/l. The mean patient age was 65.44 ± 9.94 years. The platelet was higher in patients with TBA levels below 3.5 *μ*mol/l than those in patients with TBA levels above 3.5 *μ*mol/l. The subsequent revascularization rates were not different in three groups. No significant difference in other factors and compliance with medication were observed amongst the three groups (all *p* > 0.05). Compliance with medication among CTO-PCI patients during follow-up including aspirin (72.6%), clopidogrel (38.7%), statin (61.0%), angiotensin-converting enzyme inhibition/angiotensin receptor blocker (27.9%), *β*-blocker (53.5%), and calcium channel blocker (21.5%).

The associations of TBA with conventional cardiovascular risk factors are displayed in Supplemental Table 1. Patients at older ages (*β* = 0.082, *p* = 0.042) had higher TBA than their counterparts, whereas more platelet (*β* = −0.111, *p* = 0.006) had lower TBA than their counterparts.

### 3.2. Clinical Outcomes

The median duration of follow-up was 33.5 months (interquartile range, 20.21 to 44.85 months) in three groups. During follow-up, 128(20.9%) experienced the adverse cardiovascular events, including 56 (9.1%) all-cause death, 27 (4.4%) nonfatal acute myocardial infarction, 17 (2.8%) revascularization, and 28 (4.6%) stroke.

**Figure 2:**
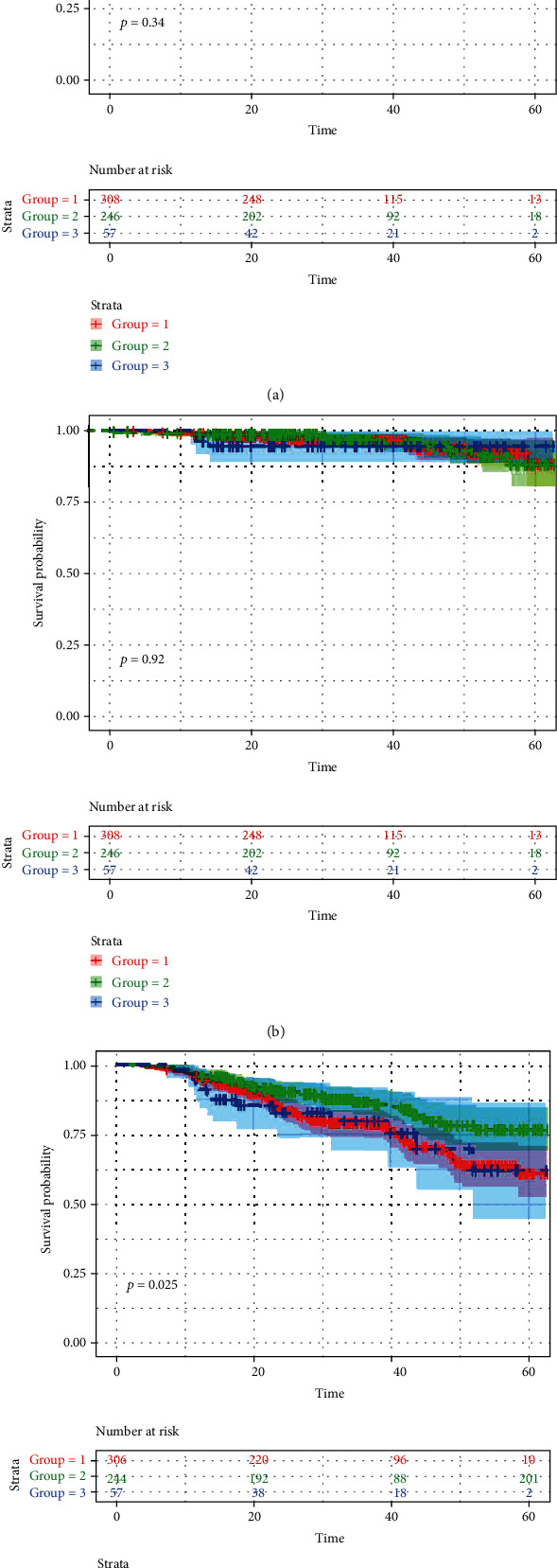
Kaplan-Meier curve of clinical outcomes judging by TBA levels. Log-rank test was used. (a) Kaplan-Meier survival curve for all-cause mortality judging by TBA levels. (b) Kaplan-Meier survival curve for cardiovascular death judging by TBA levels. (c) Kaplan-Meier survival curve for MACE judging by TBA levels. TBA: total bile acid. Group1: ~3.5, Group2: 3.5 ~ 10, Group3: ~10. MACE: major adverse cardiovascular events.

As demonstrated in the Kaplan-Meier plots, the analysis revealed no significant difference with regard to different TBA levels for the all-cause mortality (log-rank test, *p* = 0.340) and cardiovascular death (log-rank test, *p* = 0.920). Interestingly, patients with 3.5 ~ 10 *μ*mol/l TBA levels showed better prognosis than the other groups for MACE (log-rank test, *p* = 0.025) as shown in Figure 2. In Cox regression analysis, patients with 3.5 ~ 10 *μ*mol/l TBA levels had lower risk of having MACE (hazard ratio (HR): 0.60; 95% CI: 0.40 to 0.88; *p* = 0.010) as compared to ~3.5 *μ*mol/l group. There was no difference between patients with ~10 *μ*mol/l and~3.5 *μ*mol/l groups. Age (HR: 1.02; 95% CI: 1.00 to 1.04; *p* = 0.022) was significant predictors of MACE in the univariate survival analysis. In the multivariate analysis, age (HR: 1.02; 95% CI: 1.00 to 1.04; *p* = 0.021) and 3.5 ~ 10 *μ*mol/l TBA levels (HR: 0.59; 95% CI: 0.40 to 0.88; *p* = 0.009) remained independent predictors for MACE. The results are summarized in Table 2. Additionally, the results of Cox analyses for all-cause death and cardiovascular death are provided in Supplemental Table 2 and Table 3.

**Figure 3:**
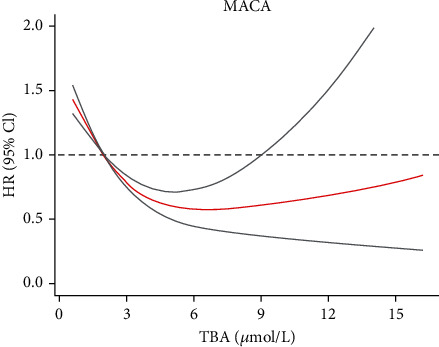
Restricted spline curves for the associations between TBA and MACE in CTO patients undergoing PCI. Red lines represent the hazard ratio, and gray lines represent the 95% confidence intervals. HR (95% CI) was adjusted by age, sex, smoking, hypertension, diabetes mellitus, LVEF, pro-BNP, heart rate, creatinine, revascularization, cholesterol, and triglyceride. TBA: total bile acid; MACE: major adverse cardiovascular events; CTO: coronary chronic total occlusion; PCI: percutaneous coronary intervention.

We used restricted cubic splines to further explore the associations of TBA, which was treated as a continuous variable, with the HR of MACE after adjusting for age, sex, smoking, hypertension, diabetes mellitus, heart rate, creatinine, revascularization, LVEF, pro-BNP, cholesterol, and triglyceride (Figure 3). An L-shaped relationship between the HR of MACE and TBA was indicated in CTO-PCI populations. The HR decreased sharply until it reached approximately 6-9 *μ*mol/l; thereafter, it tended to increase slowly. The restricted cubic splines of all-cause death and cardiovascular death are showed in Supplemental Figure 1.

### 3.3. Cross-Section Comparison

Myocardial infarction and cerebral infarction are the major disease burden with high incidence, high disability, and high mortality rate. Therefore, we studied the relationship between TBA and myocardial infarction and cerebral infarction in the cross-sectional study. 7928 patients were analyzed. Among them, 475 patients were diagnosed as myocardial infarction and 1307 were cerebral infarction. All patients were divided into three groups according to the TBA concentrations as above mentioned (≤3.5 *μ*mol/l, 3.5 ~ 10 *μ*mol/l, and ≥10 *μ*mol/l). The proportions of myocardial infarction and cerebral infarction were decreased as TBA level increased (21.3% vs. 18.7% vs. 16.1%, *p* = 0.018; 7.2% vs. 5.9% vs. 4.3%, *p* = 0.009).

In the logistic regression analysis, TBA level showed the protective role on myocardial infarction and cerebral infarction (odds ratio (OR) < 1, *p* < 0.05, as shown in Supplemental Table 4). Spearman correlation analyses showed negative correlations of LDL with TBA (*r* = −0.034, *p* = 0.004). The negative correlations of total cholesterol and TBA were marginal (*r* = −0.019, *p* = 0.092).

## 4. Discussion

The most prevalent cardiovascular diseases, especially CAD, represents the leading cause of death in the worldwide, sparing no nation, ethnicity, or economic stratum [10]. CTO is a special lesion type of CAD and is commonly encountered during coronary angiography, and PCI is a common revascularization strategy in the management of CTO [11–13]. Although genetic and other health conditions are intimately involved, the diet-gut microbiome interactions are increasingly recognized for their contribution to CAD development and progression [14]. BA as a metabolite of cholesterol and gut microbiota influences the host metabolism largely [3, 15]. Previous cross-sectional study indicated that fasting serum TBA level is associated with the presence and severity of CAD [6]. In addition, several studies revealed the reduced BA level in feces, possibly leading to AS progression [4, 16], which indicated a protective role of BA against the development of CAD. The current study further explored the predictive value of TBA for the prognosis of CTO patients undergoing PCI. The results showed that moderate fasting TBA level had a predictive value for MACE in CTO patients undergoing PCI. To the best of the authors' knowledge, this is the first study to investigate the prognostic significance of serum TBA in CTO-PCI patients with a median follow-up of 33.5 months.

It is known that cholesterol is a classic risk factor for CAD. Cholesterol is mainly eliminated from the body in the form of BAs via the liver [17]. It is reasonable to speculate that reducing the ability of cholesterol to convert to BAs will lead to cholesterol overload in the body, which develops into atherosclerosis [18]. Studies in animals have shown that mice and rats do not develop experimental atherosclerosis despite a high cholesterol diet [19–21]. They were able to cope with excess cholesterol intake by secreting large amounts of BAs. Therefore, the increase of BAs partly indicates the increase of cholesterol excretion pathway and then alleviates CAD. Furthermore, the neutral pathway accounts for at least 75% of BA production and is initiated by 7-*α*-hydroxylation of cholesterol catalyzed by cholesterol 7-*α*-hydroxylase under normal conditions [22]. Given the fact that 7-*α*-hydroxylase clears cholesterol from the plasma and the intracellular compartment, the possible mechanism for the reduction in adverse events in patients with higher TBA can be explained mainly by the increased activity of 7-*α*-hydroxylase. Moreover, studies suggested that non-CAD patients had increased activity of 7-*α*-hydroxylase; in contrast, CAD patients are unable to effectively increase the activity and concentration of 7-*α*-hydroxylase [4]. TBA had a significant negative correlation with both LDL and cholesterol level in patients with angina-like symptoms [6]. The present cross-section study also showed the negative correlation between TBA and LDL. However, TBA was not related to blood lipid in our follow-up study. The possible reason for this difference is that CTO is a kind of advanced atherosclerotic disease, usually accompanied by calcification and fibrosis, the formation of cholesterol-containing lipid cores is no longer its main pathological manifestation at this time.

Gut microbiota plays a key role in the enterohepatic cycle of BAs. Moreover, the relationship between gut microbiota, blood lipid, and atherosclerosis has been reported. The microorganism in oral and intestinal tract have been found to be associated with plasma cholesterol levels, which may be used as biomarkers of disease [23]. Compared with ApoE^−/−^ mice in specific pathogen free environment, ApoE^−/−^ mice in germ-free environment have higher cholesterol levels and lesions. Therefore, intestinal microbial mechanisms may be involved in the regulation of cholesterol and the development of atherosclerosis [24]. In addition, gut microbiota in patients with CAD differed from that in healthy individuals, which also contributed to the development of CAD through interaction with BAs [25]. Resveratrol attenuates TMAO-induced atherosclerosis by regulating BA metabolism via remodeling of the gut microbiota [26]. Moreover, BA is associated with vascular calcification and fibrosis by regulating various signaling pathways [27]. Thus, BAs may participate in different stages of atherosclerosis and affect the prognosis of CTO-PCI patients.

Platelets have various functions in physiology. The platelet membrane microparticles have multiple functions and contribute to thrombus and foam cell formation; they are involved in atherosclerotic processes, blood vessel activation, and inflammation [28]. Thus, inhibiting platelet aggregation can protect against CAD development that affects millions of people. Platelets play a significant role in the development and progression of atherosclerosis [29]. Baseline characteristics indicated that the platelet counts were lower in patients with higher TBA levels. TBA had a significant negative correlation with platelet counts in CTO-PCI patients. Thus, the associations between serum TBA and the presence of MACE may partially be attributed to the change of platelet in these patients. It is noteworthy to point out that TBA levels had not predictive effect on death. MACE in our study includes all-cause death, nonfatal acute myocardial infarction, revascularization and stroke, highlighting the intricate relationship between serum TBA and prognosis in CTO-PCI patients. Platelets play an important role in the incidence and development of acute myocardial infarction, revascularization, and stroke, which may lead to or partially explain the divergence in TBA's prediction of death and MACE. The specific reasons need to be further explored.

The present study had several limitations. First, this was a cohort study in a single center. Further multicenter and larger-scale surveys are required to confirm the results from this study and to elucidate the precise mechanisms. Second, only serum TBA was measured in our study, and specific components of bile acids were not assessed. Further prospective studies are needed to support our findings. Third, the TBA values were evaluated only once in the present study, and their changes over time during the follow-up period were not assessed. Moreover, the study only included CTO patients undergoing PCI, which suggests that the study results may not be extended to all CAD patients.

## Figures and Tables

**Table 1:** Characteristics of patients according to TBA levels.

Variables	≤3.5 (*n* = 309)	3.5-10 (*n* = 247)	≥10 (*n* = 57)	*p* value
Age (years)	65.07 ± 10.21	65.49 ± 9.64	67.19 ± 9.76	0.148
Male (%)	262 (84.8)	209 (84.6)	47 (82.5)	0.903
BMI (kg/m^2^)	24.59 ± 3.30	24.68 ± 3.61	23.83 ± 2.62	0.791
Smoking (%)	153 (49.5)	126 (51.0)	27 (47.4)	0.866
Drinking (%)	70 (22.7)	51 (20.6)	12 (21.1)	0.843
Hypertension (%)	170 (55.0)	133 (53.8)	35 (61.4)	0.585
Diabetes mellitus (%)	112 (36.2)	87 (31.5)	21 (40.7)	0.957
Prior MI (%)	98 (31.7)	80 (32.4)	19 (33.3)	0.966
DBP (mmHg)	72.0 (66.0, 80.0)	71.0 (67.0, 80.0)	73.0 (69.5, 80.0)	0.516
SBP (mmHg)	125.0 (117.0, 138.0)	125.0 (116.0, 139.0)	130.0 (117.5, 135.0)	0.916
Heart rate (bmp)	70.0 (66.0, 74.0)	70.0 (65.0, 74.0)	70.0 (67.0, 74.0)	0.201
Stent numbers	2.0 (2.0, 3.0)	2.0 (2.0, 3.0)	2.0 (2.0, 3.0)	0.412
Creatinine (mg/dl)	68.00 (57.75, 81.50)	69.60 (60.02, 79.70)	63.00 (57.75, 75.00)	0.083
hs-CRP (*μ*g/ml)	1.40 (1.20, 1.80)	1.40 (1.00, 1.60)	1.40 (1.40, 2.25)	0.133
Triglyceride (mmol/l)	1.41 (1.04, 1.96)	1.42 (0.97, 2.19)	1.41 (0.85, 2.28)	0.987
Cholesterol (mmol/l)	3.71 (3.08, 4.30)	3.67 (3.07, 4.44)	3.43 (2.96, 3.99)	0.214
LDL (mmol/l)	2.09 (1.63, 2.61)	2.09 (1.58, 2.75)	1.96 (1.49, 2.40)	0.249
HDL (mmol/l)	0.91 (0.78, 1.06)	0.94 (0.80, 1.09)	0.89 (0.77, 1.01)	0.323
Albumin (g/l)	39.15 (36.70, 41.90)	39.40 (37.00, 41.80)	38.50 (36.90, 41.50)	0.534
Platelets (10^9^/l)	184.00 (153.00, 221.50)	176.00 (141.75, 225.50)	162.00 (135.00,1 99.00)^∗^^**#**^	0.017
LVEF (%)	60.00 (45.00, 68.00)	58.00 (44.00, 66.00)	64.00 (52.00, 67.00)	0.054
CK-MB (U/l)	12.00 (9.40, 16.00)	12.00 (9.16, 15.00)	13.10 (9.00, 16.80)	0.605
pro-BNP (pg/ml)	355.0 (166.0, 1063.0)	355.0 (146.0, 1183.0)	334.00 (134.50,613.00)	0.416
TSH (uIU/ml)	1.95 (1.25, 3.20)	2.13 (1.31, 3.31)	2.12 (1.30, 2.93)	0.696
Revascularization (%)	221 (71.5)	171 (69.2)	38 (66.7)	0.702
Aspirin (%)	231 (74.8)	168 (68.0)	46 (80.7)	0.162
Clopidogrel (%)	114 (36.9)	100 (40.5)	23 (40.4)	0.284
Statin (%)	192 (62.1)	149 (60.3)	33 (57.9)	0.629
ACEI/ARB (%)	93 (30.1)	63 (25.5)	15 (26.3)	0.488
*β*-Blocker (%)	172 (55.7)	127 (51.4)	29 (50.9)	0.370
CCB (%)	65 (21.0)	57 (23.1)	10 (17.5)	0.418

BMI: body mass index; MI: myocardial infarction; DBP: diastolic blood pressure; SBP: systolic blood pressure; hs-CRP: high-sensitivity C-reactive protein; LDL: low-density lipoprotein; HDL: high-density lipoprotein; LVEF: left ventricular ejection fraction; CK-MB: creatine kinase isoenzymes MB; pro-BNP: pro-B-type natriuretic peptide; TSH: thyroid-stimulating hormone; ACEI: angiotensin-converting enzyme inhibition; ARB: angiotensin receptor blocker. CCB: calcium channel blocker. Compliance with medication is indicated. “^∗^” represents Group 1 vs. Group 3; “^**#**^” represents Group 2 vs. Group 3; Group1: ~3.5, Group2: 3.5 ~ 10, Group3: ~10.

**Table 2:** Cox proportional hazard analyses of MACE.

	Univariate analysis	*p* value	Multivariate analysis	*p* value
~3.5				
3.5-10	0.60 (0.40-0.88)	0.010	0.59 (0.40-0.88)	0.009
10~	1.05 (0.58-1.89)	0.884	1.03 (0.57-1.86)	0.923
Age	1.02 (1.00-1.04)	0.022	1.02 (1.00-1.04)	0.021
Sex	1.03 (0.64-1.67)	0.892		
Smoking	0.95 (0.67-1.36)	0.788		
Drunk	0.71 (0.45-1.12)	0.145		
Hypertension	0.86 (0.61-1.23)	0.421		
Diabetes	1.22 (0.85-1.76)	0.271		
Revascularization	0.79 (0.55-1.15)	0.219		
AMI history	1.05 (0.72-1.52)	0.804		
Stent numbers	1.06 (0.91-1.23)	0.463		
Cholesterol	0.97 (0.81-1.16)	0.739		
LVEF	0.99 (0.98-1.01)	0.340		
Creatinine	0.85 (0.46-1.57)	0.598		
hs-CRP	1.00 (0.96-1.04)	0.967		
TSH	1.01 (0.99-1.03)	0.441		
CK-MB	1.00 (1.00-1.00)	0.294		
SBP	1.00 (0.99-1.02)	0.480		
DBP	0.99 (0.97-1.01)	0.275		
Triglyceride	0.92 (0.78-1.10)	0.343		

CI: confidence interval; HR: hazard ratio; other abbreviations as in Table 1.

## Data Availability

The datasets analyzed during the current study are available from the corresponding author on reasonable request.

## Abbreviations

BAs: Bile acids
CAD: Coronary artery disease
CTO: Coronary chronic total occlusion
CI: Confidence interval
HR: Hazard ratio
hs-CRP: High-sensitivity C-reactive protein
HDL: High-density lipoprotein cholesterol
LDL: Low-density lipoprotein cholesterol
LVEF: Left ventricular ejection fraction
MACE: Major adverse cardiovascular events
PCI: Percutaneous coronary intervention
pro-BNP: Pro-B type natriuretic peptide
TBA: Total bile acids
TG: Triglycerides
TIMI: Thrombolysis in myocardial infarction.

## Supplementary Materials

Supplementary Materials Supplementary Figure 1: restricted spline curves for the associations between TBA all-cause mortality and cardiovascular death in CTO patients undergoing PCI. Supplementary Table 1: correlations of TBA with other factors. Supplemental Table 2: Cox proportional hazard analyses of all-cause death. Supplemental Table 3: Cox proportional hazard analyses of cardiovascular death. Supplemental Table 4: logistic regression analyses for the impact of TBA on myocardial and cerebral infarction.
