# Supplementary material for: The Association between Serum Total Bile Acid Level and Long-Term Prognosis in Patients with Coronary Chronic Total Occlusion Undergoing Percutaneous Coronary Intervention
Source: Dis Markers. 2022 Jun 23;2022:1434111. doi: 10.1155/2022/1434111 (PMC9246557; doi:10.1155/2022/1434111)
Supplement: Supplementary Materials — Supplementary Figure 1: restricted spline curves for the associations between TBA all-cause mortality and cardiovascular death in CTO patients undergoing PCI. Supplementary Table 1: correlations of TBA with other factors. Supplemental Table 2: Cox proportional hazard analyses of all-cause death. Supplemental Table 3: Cox proportional hazard analyses of cardiovascular death. Supplemental Table 4: logistic regression analyses for the impact of TBA on myocardial and cerebral infarction. [file 1434111.f1.zip › Supplemantal figure1A.pdf]

A

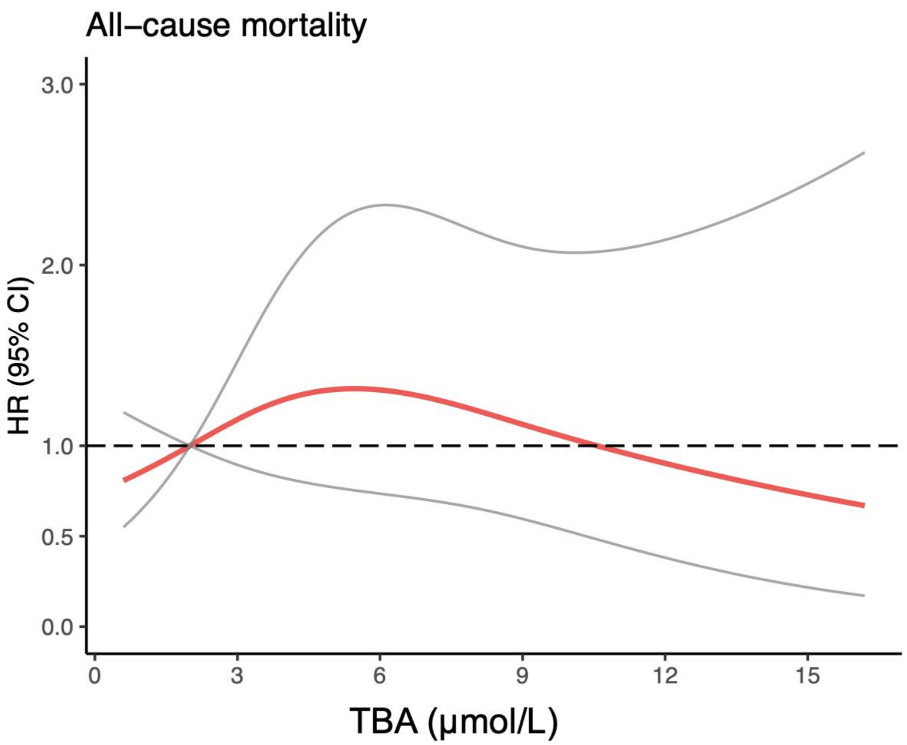

Supplemental Figure 1. Restricted spline curves for the associations between TBA (A) all-cause mortality and (B) cardiovascular death in CTO patients undergoing PCI. Red lines represent the hazard ratio, gray lines represent the 95% confidence intervals. HR (95% CI) was adjusted by age, sex, smoking, hypertension, diabetes mellitus, LVEF, pro-BNP, heart rate, creatinine, revascularization, cholesterol and triglyceride. TBA=total bile acid; CTO=coronary chronic total occlusion; PCI=percutaneous coronary intervention.
