# Supplementary material for: The Association between Serum Total Bile Acid Level and Long-Term Prognosis in Patients with Coronary Chronic Total Occlusion Undergoing Percutaneous Coronary Intervention
Source: Dis Markers. 2022 Jun 23;2022:1434111. doi: 10.1155/2022/1434111 (PMC9246557; doi:10.1155/2022/1434111)
Supplement: Supplementary Materials — Supplementary Figure 1: restricted spline curves for the associations between TBA all-cause mortality and cardiovascular death in CTO patients undergoing PCI. Supplementary Table 1: correlations of TBA with other factors. Supplemental Table 2: Cox proportional hazard analyses of all-cause death. Supplemental Table 3: Cox proportional hazard analyses of cardiovascular death. Supplemental Table 4: logistic regression analyses for the impact of TBA on myocardial and cerebral infarction. [file 1434111.f1.zip › Supplemental Tables 1-4 (1).docx]

**Supplemental Table 1. Correlations of TBA with other factors.**

|  | Coefficient | *P* value |
| --- | --- | --- |
| Age | 0.082 | 0.042 |
| LDL | -0.018 | 0.665 |
| HDL | 0.001 | 0.778 |
| Cholesterol | -0.016 | 0.685 |
| Triglycerides | 0.011 | 0.778 |
| Creatinine | -0.012 | 0.775 |
| Platelet | -0.111 | 0.006 |
| pro-BNP | -0.038 | 0.346 |
| BMI | -0.007 | 0.875 |
| LVEF | 0.019 | 0.652 |
| hs-CRP | -0.003 | 0.936 |
| Heart rate  DBP | -0.047  0.026 | 0.249  0.518 |
| SBP | 0.006 | 0.875 |
| TSH | 0.054 | 0.200 |
| Albumin | 0.022 | 0.584 |

BMI body mass index; LDL low-density lipoprotein; HDL high-density lipoprotein; hs-CRP high sensitivity C-reactive protein; LVEF left ventricular ejection fraction; pro-BNP pro-B-type natriuretic peptide; DBP diastolic blood pressure; SBP systolic blood pressure; TSH thyroid stimulating hormone.

**Supplemental Table 2. Cox Proportional Hazards Analyses of All-cause Death**

|  | Univariate analysis |  | *P* Value | Multivariate analysis | *P* Value |
| --- | --- | --- | --- | --- | --- |
| ~3.5  3.5-10  10~  Age  Sex  Smoking  Drunk  Hypertension  Diabetes  Revascularization  MI history  Stent numbers  Cholesterol  Creatinine  hsCRP  TSH  CK-MB  SBP  DBP  Triglyceride | 1.35(0.78-2.33)  0.73(0.22-2.43)  1.06(1.03-1.09)  1.04(0.51-2.12)  1.03(0.60-1.75)  1.92(0.90-4.09)  1.35(0.80-2.30)  1.25(0.73-2.14)  0.71(0.41-1.23)  1.03(0.59-1.80)  0.83(0.63-1.08)  0.87(0.66-1.16)  1.00(0.99-1.01)  1.02(0.98-1.06)  1.00(0.96-1.05)  1.00(0.99-1.01)  0.98(0.96-0.99)  0.99(0.96-1.02)  0.67(0.46-0.97) |  | 0.287  0.611  <0.001  0.919  0.923  0.090  0.265  0.419  0.219  0.907  0.163  0.341  0.151  0.382  0.959  0.415  0.031  0.401  0.035 | 1.04(1.01-1.07)  0.98(0.97-1.00)  0.88(0.63-1.21) | 0.016  0.056  0.875 |

MI myocardial infarction; CK-MB creatine kinase isoenzymes MB; hs-CRP high sensitivity C-reactive protein; DBP diastolic blood pressure; SBP systolic blood pressure; TSH thyroid stimulating hormone; CI confidence interval; HR hazard ratio. HR (95%CI) is indicated.

**Supplemental Table 3. Cox Proportional Hazards Analyses of Cardiovascular Death**

|  | Univariate analysis |  | *P* Value | Multivariate analysis | *P* Value |
| --- | --- | --- | --- | --- | --- |
| ~3.5  3.5-10  10~  Age  Sex  Smoking  Drunk  Hypertension  Diabetes  Revascularization  AMI history  Stent numbers  Cholesterol  Creatinine  hsCRP  TSH  CK-MB  SBP  DBP  Triglyceride | 1.03(0.48-2.21)  1.29(0.37-4.51)  1.05(1.01-1.09)  1.07(0.41-2.79)  1.07(0.52-2.21)  0.59(0.22-1.56)  0.76(0.37-1.56)  1.41(0.69-2.91)  0.26(0.53-1.09)  1.57(0.76-3.24)  0.95(0.69-1.31)  1.01(0.70-1.46)  1.00(0.99-1.01)  1.03(0.98-1.08)  1.01(0.98-1.05)  1.00(0.99-1.00)  0.98(0.96-1.00)  0.99(0.95-1.02)  0.74(0.46-1.17) |  | 0.938  0.687  0.014  0.898  0.846  0.286  0.457  0.349  0.082  0.221  0.751  0.944  0.301  0.251  0.485  0.115  0.104  0.478  0.194 | 1.03(0.99-1.08) | 0.176 |

Abbreviations as in Supplemental Table 2.

**Supplemental Table 4. Logistic Regression Analyses for ﻿the Impact of TBA on Myocardial and Cerebral Infarction**

|  | ≤ 3.5 ( n=3899) | 3.5-10 (n=3322) | ≥10 (n=707) |
| --- | --- | --- | --- |
| Myocardial infarction | Reference | 0.81(0.67,0.99)  *P*=0.038 | 0.59(0.40,0.88)  *P*=0.009 |
| Cerebral infarction | Reference | 0.88(0.78,0.99)  *P*=0.042 | 0.76(0.60,0.95)  *P*=0.016 |
| Myocardial & cerebral infarction | Reference | 0.85(0.76,0.95)  *P*=0.005 | 0.71(0.58,0.88)  *P*=0.001 |
