# Supplementary figures and images for: The Association between Serum Total Bile Acid Level and Long-Term Prognosis in Patients with Coronary Chronic Total Occlusion Undergoing Percutaneous Coronary Intervention
Source: Dis Markers. 2022 Jun 23;2022:1434111. doi: 10.1155/2022/1434111 (PMC9246557; doi:10.1155/2022/1434111)

B

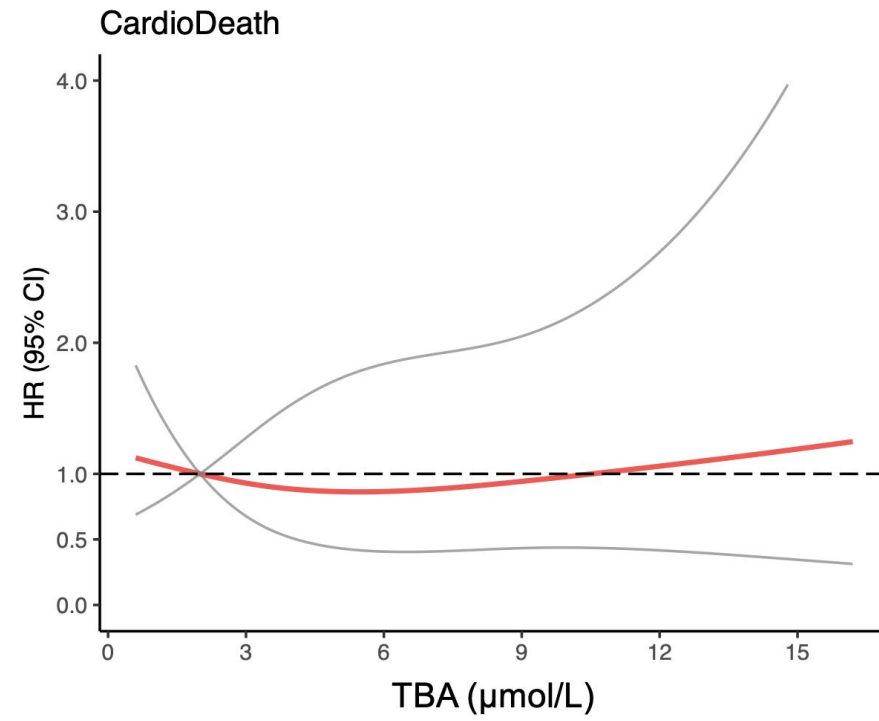

Supplement: Supplementary Materials — Supplementary Figure 1: restricted spline curves for the associations between TBA all-cause mortality and cardiovascular death in CTO patients undergoing PCI. Supplementary Table 1: correlations of TBA with other factors. Supplemental Table 2: Cox proportional hazard analyses of all-cause death. Supplemental Table 3: Cox proportional hazard analyses of cardiovascular death. Supplemental Table 4: logistic regression analyses for the impact of TBA on myocardial and cerebral infarction. [file 1434111.f1.zip › Supplemantal figure1B.pdf]
